# Serum lipoprotein(a) levels are inversely associated with metabolic dysfunction-associated steatosis liver disease progression: two cross-sectional studies and a longitudinal study

**DOI:** 10.3389/fnut.2026.1722393

**Published:** 2026-03-17

**Authors:** Wen Guo, Fei Lin, Chengxiao Yu, Jing Lu, Pei Qin, Xin Zhao, Xiaona Li, Qun Zhang

**Affiliations:** 1 Department of Health Promotion Center, The First Affiliated Hospital with Nanjing Medical University, Nanjing, China; 2 Department of Epidemiology, School of Public Health, Nanjing Medical University, Nanjing, China

**Keywords:** lipoprotein(a), metabolic dysfunction-associated steatotic liver disease, progression, regression, disease dynamics

## Abstract

**Background and aim:**

Given that abnormal lipid metabolism is a hallmark of metabolic dysfunction-associated steatotic liver disease (MASLD), this study seeks to investigate the relationship between serum lipoprotein(a) [Lp(a)] levels and the progression or regression of MASLD.

**Methods:**

A total of 12,962 participants undergoing transient elastography at the Health Promotion Center of the First Affiliated Hospital of Nanjing Medical University were included in the first cross-sectional study (Study 1). The longitudinal study (Study 2) included 17,661 individuals from the same center, each with at least two health check-ups involving abdominal ultrasonography. Another cross-sectional study (Study 3) included 5,927 individuals from the UK Biobank cohort who had undergone both magnetic resonance imaging proton density fat fraction (MRI-PDFF) and Lp(a) testing.

**Results:**

Cross-sectional analysis (Study 1) revealed that elevated Lp(a) levels were inversely correlated with the severity of both hepatic steatosis and fibrosis. Longitudinal data (Study 2) further demonstrated that baseline serum Lp(a) levels were decreased in participants with the incident of MASLD, while increased in participants with the regression of MASLD during the follow-up period. A lower baseline Lp(a) level was an independent factor for new-onset MASLD and non-regression of MASLD: the fully adjusted hazard ratios (HR) were 0.895 (95%CI 0.834–0.962, *p* < 0.001) and 0.889 (95%CI 0.8110.975, *p* = 0.012), respectively. In study 3, serum Lp(a) levels were negatively correlated with MASLD (OR = 0.885, 95% CI 0.746–0.980, *p* = 0.025). Notably, restricted cubic spline analysis revealed a significant linear dose–response relationship between serum Lp(a) levels and MASLD transitions.

**Conclusion:**

Serum Lp(a) levels are inversely associated with both the progression and regression of MASLD, indicating its potential role in reflecting disease dynamics.

## 1 Introduction

Metabolic dysfunction-associated steatotic liver disease (MASLD) has become a growing global health concern, with its prevalence rising worldwide. Owing to its progression potential toward metabolic dysfunction-associated steatohepatitis (MASH),

hepatic fibrosis, liver cirrhosis, even to hepatocellular carcinoma, MASLD is emerging as the most prevalent chronic liver disease (1). MASLD extends beyond localized liver damage to produce significant systemic effects, which drive the development of comorbidities such as cardiovascular disease, type 2 diabetes mellitus (T2DM), chronic kidney disease, and even extrahepatic tumors (2, 3). Given the paucity of available treatments for MASLD and its potential for devastating long-term health consequences, it is crucial to comprehend its underlying risk factors and pathogenesis and then early intervention.

Lipoprotein(a) (Lp[a]) is an atherogenic lipoprotein composed of an LDL-like particle covalently linked to a unique apolipoprotein(a) chain. A growing number of studies have indicated that high levels of Lp(a) increased risk of cardiovascular disease (CVD), even with optimal low-density lipoprotein cholesterol (LDL-C) management (4, 5). In recent years, the link between serum Lp(a) levels and metabolic disorders, including MASLD has attracted attention. Elevated Lp(a) levels were positively associated with a higher risk of left ventricular hypertrophy in a cross-sectional study of 2,096 new-onset myocardial infarction patients (6) and was also linked to an increased risk of panvascular disease (7). Multiple cross-sectional studies have reported an inverse association between Lp(a) levels and liver-related conditions. An analysis of 2,308 participants from NHANES III demonstrated a significant inverse linear relationship between Lp(a) and the risk of non-alcoholic fatty liver disease (NAFLD) (8). Furthermore, a study from Colombia revealed that Lp(a) levels were inversely correlated with the Fibrosis-4 (FIB-4) score, a marker of liver fibrosis severity in patients with MASLD (9). Additionally, a Chinese study identified that among patients with T2DM, the combination of a low adipose insulin resistance index and high Lp(a) levels was associated with the lowest risk of MASLD (10). However, the above studies were limited by small sample sizes and cross-sectional designs and did not assess the association of Lp(a) with hepatic steatosis or fibrosis severity. Emerging evidence from the UK Biobank prospective study suggests that Low Lp(a) levels confer an increased risk of MASLD, cirrhosis, and hepatocellular carcinoma (11). Lp(a) levels are primarily genetically determined and vary in different ethnic groups. Up to date, longitudinal data on the association between Lp(a) levels and disease transition in Chinese populations remain unavailable.

Although liver biopsy is the established gold standard for the diagnosis of MASLD and MASLD-related advanced fibrosis, its invasive nature and high cost restrict routine clinical use, especially in individuals undergoing routine health examinations. Magnetic resonance imaging proton density fat fraction (MRI-PDFF) is established as the non-invasive gold standard for diagnosing hepatic steatosis, offering an accurate and precise method for its detection (12). Liver transient elastography is a guideline-recommended noninvasive tool for assessing hepatic steatosis and fibrosis, demonstrating excellent correlation with liver biopsy (13). To precisely and comprehensively evaluate the relationship between Lp(a) and MASLD, this study was designed with three separate analyses. First, a cross-sectional cohort in a Chinese population was established using liver transient elastography to assess hepatic steatosis and fibrosis, aiming to investigate the association of Lp(a) with the severity of hepatic steatosis or fibrosis (Study 1). Second, a prospective cohort n a Chinese population was designed to longitudinally assess the role of Lp(a) in the progression and regression of MASLD over time (Study 2). Finally, an independent cross-sectional analysis of the UK Biobank employed MRI-PDFF for precise hepatic fat quantification to further examine the relationship between Lp(a) and MASLD (Study 3).

## 2 Methods

### 2.1 Study population

The current study included three distinct cohort studies. Study 1 enrolled 13,870 participants who underwent transient elastography during routine health examinations at the Health Promotion Center of the First Affiliated Hospital of Nanjing Medical University between October 2017 and April 2024. Study 2 also recruited 18,101 participants from the same center as study 1, who underwent at least two health check-ups including abdominal ultrasound between January 2017 and January 2021. For Study 3, participants from the UK Biobank (under application number 85248) who had undergone both MRI-PDFF and Lp(a) testing were included in the analysis. Exclusion criteria included: excessive alcohol consumption (defined as ≥30 g/d for men and ≥20 g/d for women); history of hepatitis, cirrhosis, or other chronic liver diseases; history of cancer, coronary heart disease, kidney disorders, or a history of lipid-lowering medication use. Additionally, participants with related missing data such as body mass index (BMI), systolic blood pressure (SBP), diastolic blood pressure (DBP), fasting plasma glucose (FPG), lipid profiles or liver function were further excluded. At the end, 12,962 participants were enrolled in study 1, and 17,661 in study 2. 5,937 eligible participants were included in study 3. Ethical approval for study 1 and study 2 was granted by the Human Research Ethics Committee of the First Affiliated Hospital of Nanjing Medical University (2023-SR-410). As the research involved a retrospective analysis of anonymized routine health screening data, the requirement for obtaining informed consent was waived. The UK Biobank received ethical approval from the North West Multi-Centre Research Ethics Committee (REC reference: 14/NW/0157). All participants provided informed consent to participate.

### 2.2 Data collection in study 1 and study 2

Demographic characteristics (age and sex), medical history, and smoking status were assessed by physicians using interviewer-administered questionnaires. Anthropometric parameters including weight, height, SBP and DBP were measured by trained nurses according to a standardized protocol. Measurements of plasma glucose, lipid concentrations, liver function and serum uric acid were performed enzymatically on an AU5800 automatic biochemical analyzer (Olympus Corporation, Tokyo, Japan). Lp(a) levels were measured using an immunonephelometry method. Glycosylated hemoglobin A1c (HbA1c) were quantified using an immunoturbidimetric assay performed on a Cobra Integra 800 automatic analyzer (Roche Diagnostics, Basel, Switzerland).

### 2.3 Measurement of Lp(a) levels in study 3

Lp(a) levels were measured by immunoturbidimetry using the Randox assay (Data Field 30,790). The assay range was 5.76 to 189 nmol/L. In the present study, Lp(a) concentrations were converted using the standard factor (1 mg/dL = 2.15nmo/L) as referenced to the previous study (11).

### 2.4 Definition of the severity of hepatic steatosis and liver fibrosis

MASLD was diagnosed based on the presence of hepatic steatosis along with at least one cardiometabolic risk factor which was referred as previous studies (15).

In study 1, the degree of hepatic steatosis and liver fibrosis were evaluated by transient elastography using a FibroTouch FT100 system (Wuxi Hisky Medical Tech. Co., Ltd., China). The cut-off values of the degree of hepatic steatosis and liver fibrosis were referred to the previous study (16). Hepatic steatosis was classified into three categories based on the fat attenuation parameter (FAP) values: non-MASLD (*n* = 4,105), mild MASLD (*n* = 1,919), moderate (*n* = 6,698) and severe (*n* = 240). Liver fibrosis was classified into four categories based on the liver stiffness measurement (LSM) values: F0–F1 (*n* = 6,938), F2 (*n* = 1,343), F3 (*n* = 392) and F4 (*n* = 184).

In study 2, each participant underwent a minimum of two abdominal ultrasound examinations (Siemens Acuson X300, German). We then classified participants in 4 groups with respect to fatty status: stable non-MASLD (*n* = 9,737), developed to MASLD (*n* = 4,854), regressed to non-MASLD (*n* = 858) and stable MASLD (*n* = 2,212). The definition of four groups was referred as previous studies (17, 18).

In study 3, hepatic steatosis was quantified by MRI-PDFF using a Siemens Healthineers system (Erlangen, Germany), with a threshold of ≥5% applied for diagnosis.

### 2.5 Statistical analysis

Statistical analysis was performed in SPSS 23.0 statistics software and R version 3.3.0 software. Continuous variables were presented as the mean with standard deviation or median (interquartile range) depending on variable distribution, while categorical variables were expressed as numbers (n) with proportions (%). Intergroup comparisons were conducted using Student’s t-test, one-way ANOVA, Mann–Whitney U test, or Pearson’s chi-square test, as appropriate. Multivariate logistic regression or Cox regression analyses were performed to identify significant determinants, with adjustment for potential confounders based on univariate results and literature reports. The adjusted covariates included age, sex, smoking status, BMI, hypertension, diabetes, dyslipidemia, and serum uric acid. Restricted cubic spline (RCS) curves adjusted for the same variables as in adjusted model were conducted to evaluate the potential linear or nonlinear dose–response associations. Statistical significance was set at *p*-value <0.05 (two-tailed).

## 3 Results

### 3.1 Study 1: Cross-sectional association of Lp(a) levels with the severity of MASLD assessed by liver transient elastography in a Chinese population

Demographic and clinical characteristics of participants by MASLD status are presented in Table 1. Individuals with MASLD showed a higher proportion of smokers, and metabolically unhealthy (higher BMI, SBP, DBP, total cholesterol (TC), triglyceride (TG), low-density lipoprotein cholesterol (LDL-C), FPG, HbA1c and uric acid). Moreover, were also more frequently of male gender than those without MASLD.

**Table 1:** Baseline characteristics of individuals with or without MASLD in study 1.

Characteristics	Non-MASLD (*n* = 4,105)	MASLD (*n* = 8,857)	*P*
Age (years)	48.74 ± 10.86	48.74 ± 10.61	0.999
Male (*n*, %)	1,788 (43.6)	6,372 (71.9)	<0.001
Smoking (*n*, %)	636 (15.5)	2,411 (27.2)	<0.001
Diabetes (*n*, %)	212 (5.2)	1,166 (13.2)	<0.001
Hypertension (*n*, %)	368 (9.0)	1,609 (18.2)	<0.001
Dyslipidemia (*n*, %)	983 (23.9)	4,076 (46.0)	<0.001
BMI (kg/m^2^)	22.78 ± 2.29	26.31 ± 3.26	<0.001
SBP (mmHg)	122.55 ± 17.34	129.29 ± 16.84	<0.001
DBP (mmHg)	74.64 ± 10.76	80.11 ± 11.10	<0.001
WBCC (×10^9^/L)	5.43 ± 2.00	5.95 ± 1.51	<0.001
Monocyte count (×10^9^/L)	0.37 ± 0.12	0.41 ± 0.13	<0.001
Neutrophil count (×10^9^/L)	3.16 ± 1.24	3.44 ± 1.14	<0.001
FPG (mmol/L)	5.21 ± 0.99	5.64 ± 1.40	<0.001
HbA1c (%)	5.46 ± 0.62	5.70 ± 0.83	<0.001
TC (mmol/L)	5.16 ± 0.99	5.30 ± 1.07	<0.001
TG (mmol/L)	1.29 ± 0.87	2.06 ± 1.59	<0.001
LDL-C (mmol/L)	3.16 ± 0.72	3.32 ± 0.75	<0.001
HDL-C (mmol/L)	1.45 ± 0.32	1.26 ± 0.28	<0.001
Uric acid (umol/L)	308.35 ± 78.74	368.54 ± 89.66	<0.001
ALT (U/L)	16.9 (12.8–23.1)	24.5 (17.4–36.9)	<0.001
AST (U/L)	20.9 (18.0–25.1)	23.2 (19.4–28.6)	<0.001
GGT (U/L)	18.9 (14.0–28.0)	31.9 (20.9–51.9)	<0.001

Values are presented as mean ± standard deviation, *n* (%), or median (interquartile range).

BMI, body mass index; SBP, systolic blood pressure; DBP, diastolic blood pressure; WBCC, white blood cell count; FPG, fasting plasma glucose; TC, total cholesterol; TG, triglyceride; LDL-C, low-density lipoprotein cholesterol; HDL-C, high-density lipoprotein cholesterol; ALT, alanine aminotransferase; AST, aspartate transaminase; GGT, gamma-glutamyl transpeptidase; MASLD, metabolic dysfunction-associated steatotic liver disease.

The distributions of the Lp(a) levels stratified by the severity of hepatic steatosis are shown in Supplementary Figure 1A. The Lp(a) levels exhibited a decreasing trend with the severity of hepatic steatosis. The Lp(a) levels decreased from F0-F1, F2, F3 to F4 (Supplementary Figure 1B).

Logistic regression analyses showed a 19.8% decreased mild MASLD risk (OR = 0.802, 95% CI 0.721–0.893, *p* < 0.001), a 34.5% decreased moderate MASLD risk (OR = 0.655, 95% CI 0.606–0.707, *p* < 0.001), and a 56.3% decreased severe MASLD risk (OR = 0.437, 95% CI 0.341–0.558, *p* < 0.001) per 1-unit increase of log_10_ Lp(a) level (Supplementary Table 1). After adjusting for potential confounders, the significant relationship between serum Lp(a) levels and moderate MASLD, as well as severe MASLD still existed. However, the relationship between serum Lp(a) levels and mild MASLD was attenuated (Supplementary Table 1).

Logistic regression analyses showed that Lp(a) levels were inversely associated with the F2 stage (OR = 0.806, 95% CI 0.717–0.907, *p* < 0.001), F3 stage (OR = 0.671, 95% CI 0.547–0.823, *p* < 0.001) and F4 stage (OR = 0.434, 95% CI 0.324–0.583, *p* < 0.001) after adjusting for potential confounders and metabolic variables (Supplementary Table 2).

**Figure 1:**
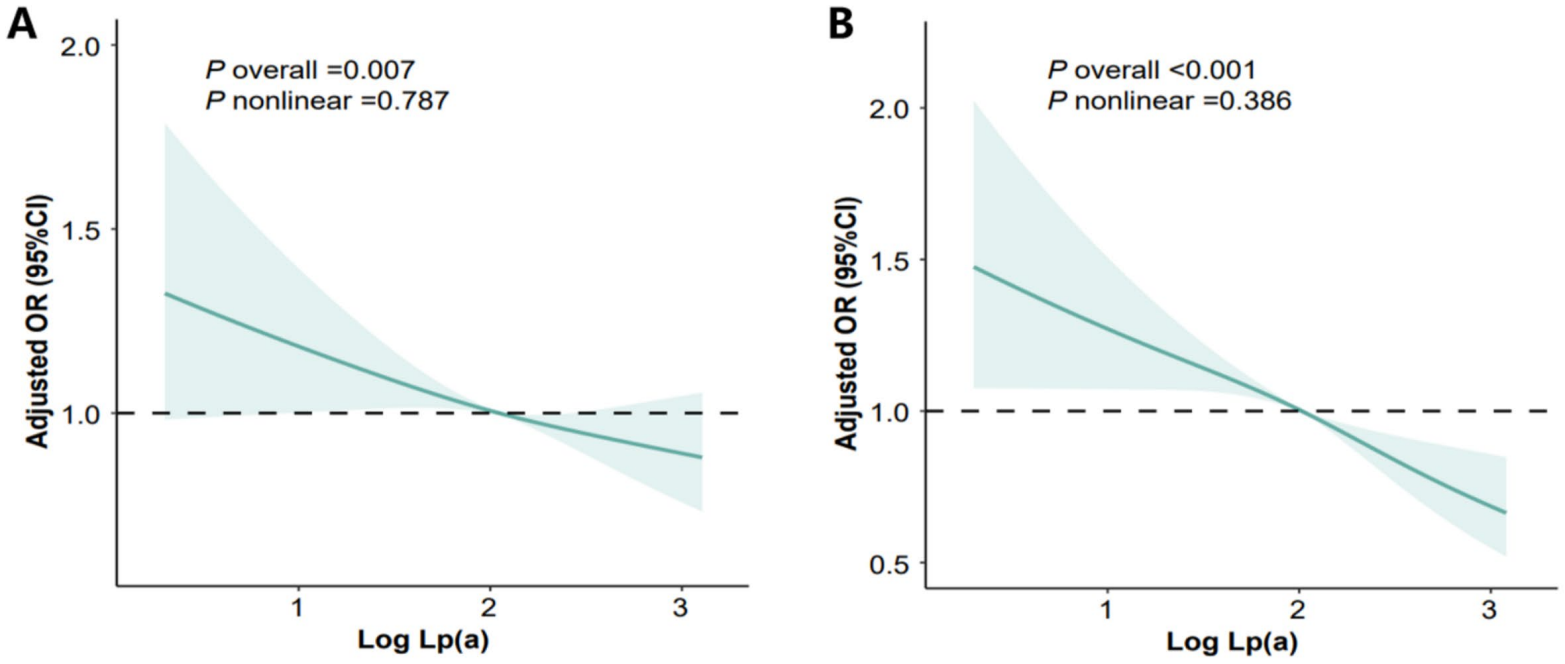
Dose-effect associations of the log_10_Lp(a) level with MASLD and liver fibrosis in study 1. **(A)** the association between the log_10_Lp(a) level with MASLD; **(B)** the association between the log_10_Lp(a) level and liver fibrosis.

After adjusting for confounders, we found that serum Lp(a) levels exhibited an independent, linear association with MASLD (*P* for nonlinear = 0.787) and liver fibrosis severity (*p* for nonlinear = 0.386) (Figures 1A,B).

### 3.2 Study 2: Prospective association of Lp(a) levels with progression or regression of MASLD

The biochemical parameters and clinical characteristics of participants in study 2 are.

shown in Table 2. Median follow-up time was 25 months. At follow-up, 9,737 participants (55.13%) remained stable non-MASLD, 4,854 (27.48%) progressed to MASLD, 858 (4.86%) regressed to non-MASLD, and 2,212 (12.53%) remained stable with MASLD. Participants with incident MASLD were more likely to have hypertension, diabetes and dyslipidemia, higher levels of uric acid, alanine aminotransferase (ALT), aspartate transaminase (AST), and gamma-glutamyl transpeptidase (GGT) at baseline compared with participants with stable non- MASLD. Participants who regressed to non-MASLD exhibited lower baseline levels of BMI, TG, uric acid, ALT, AST and GGT compared to those with stable MASLD. Notably, participants who progressed to MASLD showed a decrease in median Lp(a) levels (from 13.8 to 11.7 mg/dL, *p* < 0.001), whereas those who regressed to non-MASLD showed an increase (from 9.85 to 12.95 mg/dL, *p* < 0.001).

**Table 2:** Baseline characteristics according to the categories of MASLD progression and regression in study 2.

Characteristics	Incident MASLD		MASLD regression	
	No (*n* = 9,737)	Yes (*n* = 4,854)	No (*n* = 2,212)	Yes (*n* = 858)
Age (years)	41.15 ± 11.46	43.80 ± 10.96^*^	43.61 ± 11.32	46.30 ± 11.65^#^
Male (*n*, %)	4,246 (43.6)	56565656	1871 (84.6)	658 (76.7)^#^
Smoking (*n*, %)	1,152 (11.80)	1,120 (23.10)^*^	591 (26.7)	197 (23.0)^#^
Diabetes (*n*, %)	264 (2.7)	785 (7.9)^*^	233 (10.5)	91 (10.6)
Hypertension (*n*, %)	559 (5.7)	700 (14.4)^*^	366 (16.5)	149 (17.4)
Dyslipidemia (*n*, %)	2,017 (20.7)	2,136 (44.0)^*^	1,101 (49.8)	376 (43.8)^#^
BMI (kg/m^2^)	22.50 ± 2.71	25.38 ± 3.05^*^	26.78 ± 2.92	25.92 ± 2.73^#^
WBCC (×10^9^/L)	5.79 ± 1.42	6.23 ± 1.54^*^	6.56 ± 1.54	6.38 ± 1.52^#^
Monocyte count (×10^9^/L)	0.37 ± 0.12	0.41 ± 0.13^*^	0.42 ± 0.13	0.39 ± 0.13^#^
Neutrophil count (×10^9^/L)	3.38 ± 1.13	3.60 ± 1.19^*^	3.82 ± 1.17	3.70 ± 1.16^#^
SBP (mmHg)	119.71 ± 15.62	126.92 ± 15.79^*^	131.67 ± 15.28	131.15 ± 16.14
DBP (mmHg)	73.66 ± 10.45	79.03 ± 10.93^*^	82.75 ± 10.57	82.04 ± 11.03
FPG (mmol/L)	5.15 ± 0.76	5.50 ± 1.11^*^	5.62 ± 1.42	5.64 ± 1.27
HbA1c (%)	5.39 ± 0.45	5.57 ± 0.65^*^	5.66 ± 0.81	5.62 ± 0.72
TC (mmol/L)	5.11 ± 0.97	5.37 ± 1.02^*^	5.29 ± 1.00	5.31 ± 0.98
TG (mmol/L)	1.20 ± 0.80	1.99 ± 1.57^*^	2.20 ± 1.56	1.91 ± 1.34^#^
LDL-C (mmol/L)	3.11 ± 0.72	3.37 ± 0.74^*^	3.34 ± 0.72	3.40 ± 0.72
HDL-C (mmol/L)	1.44 ± 0.31	1.25 ± 0.27^*^	1.16 ± 0.22	1.21 ± 0.24^#^
Uric acid (umol/L)	309.29 ± 78.69	370.70 ± 84.81^*^	392.26 ± 81.58	368.37 ± 81.94^#^
ALT (U/L)	15.4 (11.6–21.6)	22.8 (16.4–33.2)^*^	29.7 (21.2–45.8)	24.4 (16.9–35.3)^#^
AST (U/L)	19.6 (16.8–23.2)	21.8 (18.4–26.4)^*^	24.4 (20.3–30.2)	22.9 (19.2–27.9)^#^
GGT (U/L)	17.5 (13.4–25.8)	28.7 (19.8–43.9)^*^	35.9 (25.0–54.7)	30.1 (21.5–49.0)^#^

Values are presented as mean ± standard deviation, *n* (%), or median (interquartile range).

BMI, body mass index; WBCC, white blood cell count; SBP, systolic blood pressure; DBP, diastolic blood pressure; FBG, fasting plasma glucose; TC, total cholesterol; TG, triglyceride; LDL-C, low-density lipoprotein cholesterol; HDL-C, high-density lipoprotein cholesterol; AL, alanine aminotransferase; AST, aspartate transaminase; GGT, gamma-glutamyl transpeptidase; MASLD, metabolic dysfunction-associated steatotic liver disease. Compared with stable non-MASLD, ^*^p < 0.05; Compared with regressed MASLD, ^#^*p* < 0.05.

**Figure 2:**
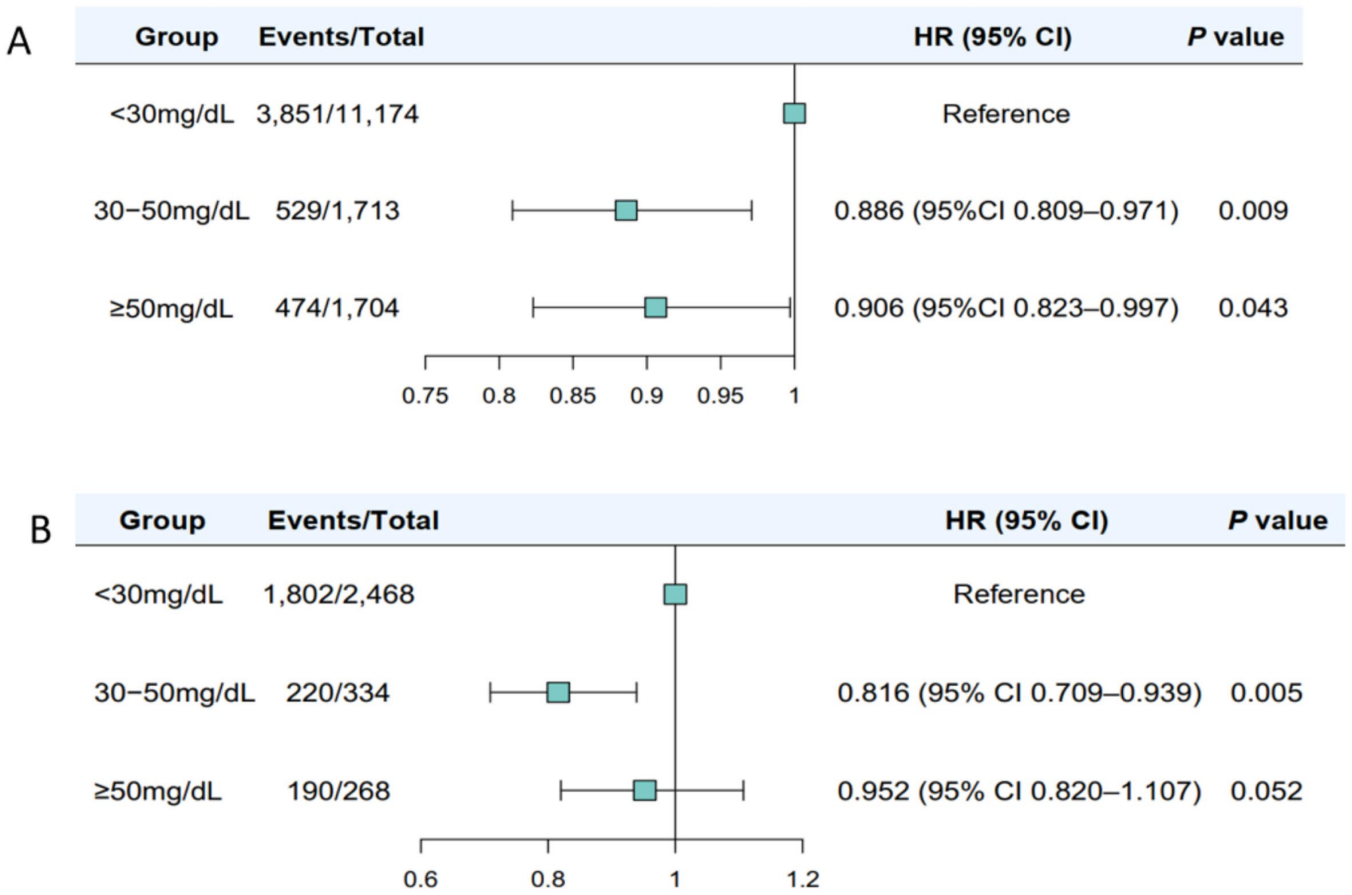
Cox proportional hazard analyses the associations of serum Lp(a) levels with the progression or regression of MASLD in study 2. **(A)** the association between serum Lp(a) levels and the progression of MASLD; **(B)** the association between serum Lp(a) levels and the regression of MASLD.

Compared with Lp(a) < 30 mg/dL, the multivariable-adjusted HRs of incident MASLD were 0.886 (95% CI 0.809–0.971, *p* = 0.009) for Lp(a) 30–50 mg/dL and 0.906 (95% CI 0.823–0.997, *p* = 0.043) for Lp(a) ≥ 50 mg/dL (Figure 2A). Moreover, the association of Lp(a) levels with incident MASLD was unchanged when modelled with continuous log_10_Lp(a) levels (per 1-unit increase) (HR = 0.878, 95% CI 0.833–0.926, *p* < 0.001). Even after adjusting for potential metabolic and clinical confounders, baseline serum Lp(a) levels remained significantly associated with stable MASLD (adjusted HR = 0.841, 95%CI 0.776–0.912, *p* < 0.001) (Figure 2B). Compared with Lp(a) < 30 mg/dL, the multivariable-adjusted HRs of stable MASLD were 0.816 (95% CI 0.709–0.939, *p* = 0.005) for Lp(a) 30–50 mg/dL and 0.952 (95% CI 0.820–1.107, *p* = 0.052) for Lp(a) ≥ 50 mg/dL.

**Figure 3:**
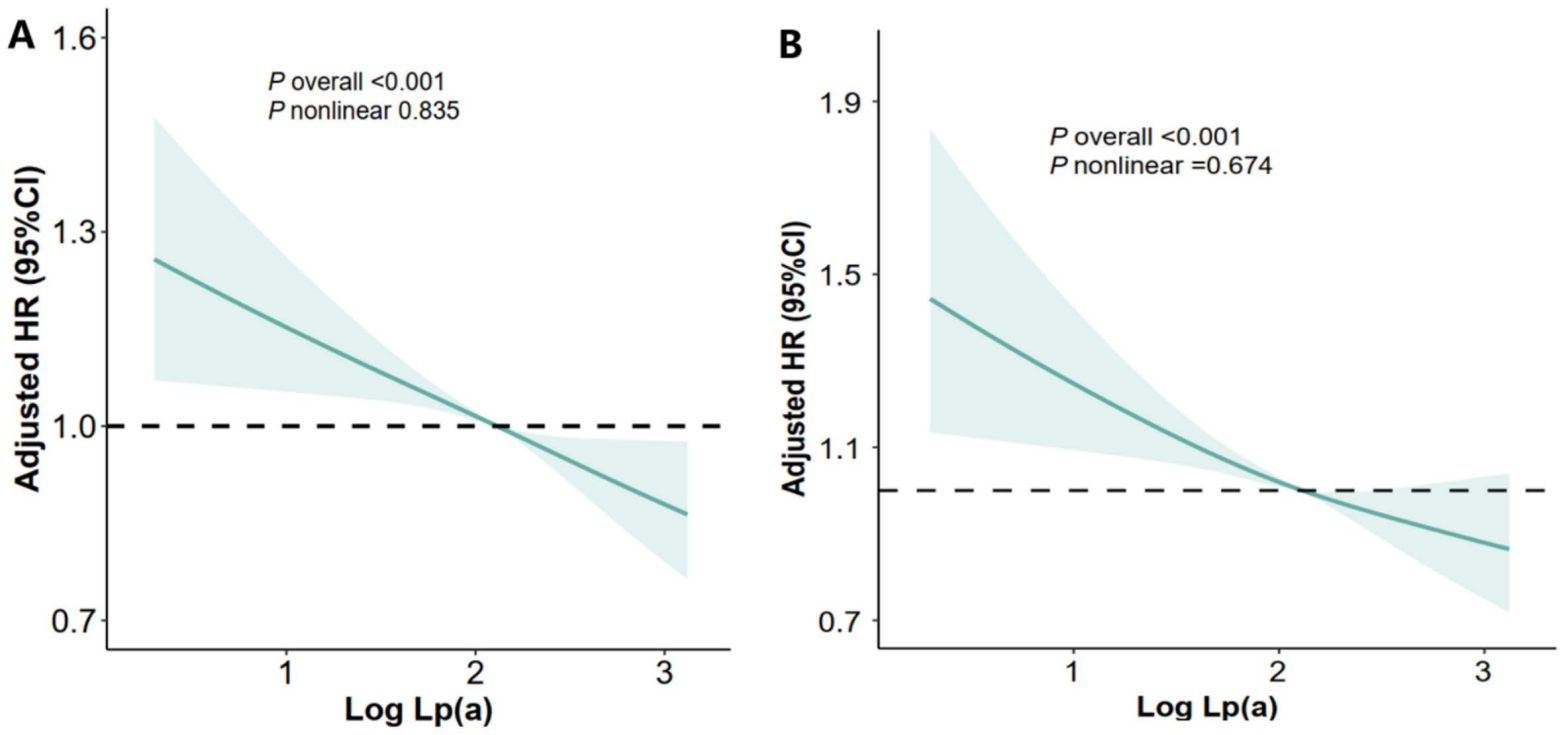
Dose-effect associations of the log_10_Lp(a) level with the progression or regression of MASLD. **(A)** the association between the log_10_Lp(a) level and the progression of MASLD; **(B)** the association between the log_10_Lp(a) level and the regression of MASLD.

Furthermore, restricted cubic spline analysis revealed a clear dose–response relationship between baseline Lp(a) levels and both MASLD progression and regression (*P* for non-linearity = 0.835 and 0.674, respectively) (Figures 3A,B).

### 3.3 Study 3: Cross-sectional association of Lp(a) levels with MASLD assessed by MRI-PDFF in the UK biobank

The detailed characteristics stratified by MASLD or not are presented in Supplementary Table 3. Median Lp(a) levels were significantly lower in the MASLD group than in the non-MASLD group (8.47 vs. 9.86 mg/dL; *p* = 0.006).

After full adjustment for potential confounders, participants in the highest (tertiles 3 of Lp(a) levels) and middle (tertiles 2 of Lp(a) levels) Lp(a) tertiles had significantly lower risks of MASLD compared to those in the lowest tertile (tertiles 1 of Lp(a) levels), with adjusted ORs of 0.819 (95% CI 0.696–0.964, *p* = 0.016) and 0.806 (95% CI 0.684–0.948, *p* = 0.009), respectively (Table 3). Moreover, the inverse association between serum Lp(a) levels and incident MASLD risk (OR = 0.885, 95% CI 0.746–0.980, *p* = 0.025) remained significant when Lp(a) was analyzed as a continuous variable (per 1-unit increase of log_10_Lp(a) level).

**Table 3:** Logistic regression models in predicting MASLD according to serum Lp(a) levels in study 3.

Parameters	Model 1		Model 2	
	OR (95%CI)	*P*	HR (95%CI)	*P*
Tertiles 1	Reference		Reference	
Tertiles 2	0.796 (0.688–0.921)	0.002	0.806 (0.684–0.948)	0.009
Tertiles 3	0.834 (0.722–0.964)	0.014	0.819 (0.696–0.964)	0.016
Per 1-unit increase of log_10_ Lp(a)	0.858 (0.759–0.970)	0.014	0.885 (0.746–0.980)	0.025

Modle1: unadjusted.

Modle2: adjustment for age, sex, smoking, BMI, hypertension, diabetes, dyslipidemia, and serum uric acid.

MASLD, metabolic dysfunction-associated steatotic liver disease; Lp(a), lipoprotein(a); BMI, body mass index; HR, hazard ratio; CI, confidence intervals.

## 4 Discussion

This study investigated the relationship between serum Lp(a) levels and metabolic MASLD using both cross-sectional and longitudinal designs. Cross-sectional analyses assessed the associations between Lp(a) levels and the severity of hepatic steatosis and fibrosis. Prospectively, the study examined whether baseline Lp(a) levels predicted the progression or regression of MASLD in a general population cohort. The results demonstrated an inverse correlation between serum Lp(a) levels and the severity of both hepatic steatosis and fibrosis. To the best of our knowledge, this represents the first longitudinal, population-based study to identify Lp(a) as a factor associated with both the progression and regression of MASLD. Finally, these findings were validated in the large, independent UK Biobank cohort. These combined results indicated that serum Lp(a) levels were closely correlated with MASLD and would serve as an indicator for MASLD progression or regression.

Lp(a) is synthesized exclusively in in the liver and consists of apolipoprotein (apo) B-100 and apolipoprotein(a) (19). Given its pro-inflammatory and pro-atherosclerotic properties, Lp(a) has been identified as one important risk factor for CVD by epidemiologic studies, mendelian randomization studies and meta-analyses, even at the normal range of LDL-C (20, 21). The role of Lp(a) in metabolic diseases has attracted growing interest. A recent cohort study supported that there was an inverse correlation between baseline Lp(a) levels and incident T2DM in patients with acute coronary syndrome (14). A subsequent study further confirmed that Lp(a) levels were inversely correlated with metabolic syndrome and its components (22). Moreover, inverse relationships were observed between Lp(a) levels and measures of adiposity, namely BMI and waist circumference (23). MASLD, formerly known as NAFLD, is a liver condition characterized by steatosis and is considered the liver-specific expression of metabolic syndrome. Several prior studies have explored possible relationships between Lp(a) levels and NAFLD or MASLD. An inverse relationship between Lp(a) levels and the presence of NAFLD was observed in a cross-sectional study conducted in Korea (24). However, this association disappeared after adjustment for homeostasis model assessment of insulin resistance. Another cross-sectional study from Japan indicated that Lp(a) levels were inversely correlated with biopsy confirmed advanced NASH (25). Besides, two cross-sectional studies demonstrated that lower serum Lp(a) levels were associated with liver fibrosis, as measured by liver biopsy or FIB-4 score (9, 26). Nevertheless, the positive link between serum Lp(a) levels and NAFLD severity was proven by a study performed in biopsy-proved NAFLD patients in China (27). In addition, a systematic review and meta-analysis proven that there was no significant difference in circulating Lp(a) levels between NAFLD patients and non-NAFLD controls when Lp(a) levels were assessed by turbidimetry (28). Due to conflicting findings on Lp(a) and NAFLD, along with limited research on Lp(a) and MASLD severity in China, our large cross-sectional study extends previous work by examining the association of Lp(a) levels with MASLD severity, as assessed by transient elastography, in a Chinese adult population. The present study demonstrated negative relationships between Lp(a) levels and the severity of both hepatic steatosis and fibrosis. Moreover, results of the present study revealed significant linear, dose–response relationships between Lp(a) levels and the risk of both MASLD and liver fibrosis. Lp(a) has been the variability in levels between different ethnic groups (29). Whether the inverse associations of Lp(a) levels with MASLD extends to other ethnic groups remains to be established. To reinforce these findings, the UK Biobank database was used to assess the link between Lp(a) levels and MASLD. The UK Biobank contains detailed, high-quality, and diverse health data on over 500,000 UK residents aged 40–69 years recruited between 2006–2010. As we know, MRI-PDFF is a noninvasive, quantitative tool to accurately measure liver fat content (30). Similarly, the present study, using UK Biobank database from an MRI-PDFF-based population, found that Lp(a) levels were inversely correlated with MASLD. Therefore, these findings suggest that Lp(a) could be a potential biomarker for the early detection of MASLD. To address this hypothesis, a longitudinal study was conducted to investigate the association between Lp(a) levels and MASLD transitions. Our findings showed that the decrease of Lp(a) levels was observed in participants who developed MASLD, while the increase of Lp(a) levels was observed in participants who regressed to non-MASLD state. The present study, the longitudinal study to date, supports the idea that an inverse association was observed between baseline Lp(a) levels and MASLD outcomes: lower levels conferred both a higher risk of incident MASLD and a lower probability of disease regression. These findings suggest that Lp(a) level may be a novel biomarker for predicting the dynamics of MASLD, namely the likelihood of disease progression or regression.

Mechanisms behind the association of Lp(a) levels with MASLD has not been fully explained yet. Lp(a) is primarily synthesized in the liver. The negative associations of Lp(a) levels with ALT, AST and GGT were observed in this study. In addition, participants with elevated ALT, AST and GGT had lower serum Lp(a) levels than those with normal ALT, AST and GGT. Taken together, it is hypothesized that the low Lp(a) levels observed in MASLD patients are hypothesized to reflect impairment of hepatic function. The catabolism of Lp(a) involves several receptors, including the LDL receptor (LDLR), VLDL receptor (VLDL-R), chylomicron remnant receptor, and scavenger receptors, as demonstrated *in vitro* (31). Notably, studies have shown that fatty liver disease is associated with an upregulation of these receptors (32, 33). It is therefore plausible that in the context of MASLD, enhanced catabolism or clearance of Lp(a) particles contributes to their lower circulating levels. Furthermore, several studies indicated that Lp(a) levels were inversely correlated with insulin resistance (34, 35). Hyperinsulinemia, a hallmark of MASLD, suppress apolipoprotein(a) synthesis in hepatocytes (36), leading to reduced circulating Lp(a) levels. Of further relevance are the findings that MASLD patients show aberrations in hormones other than insulin, such as testosterone, IFG-1, or thyroid hormones, all of which are known to impact APOA expression (37). However, the exact reasons and mechanisms need to be further explored.

A key strength of this study is the conduction of two large cross-sectional cohorts involving distinct ethnic populations to systematically examine the link between Lp(a) and MASLD. To complement the cross-sectional findings, we innovatively conducted a longitudinal study. However, several limitations should be acknowledged. First, despite extensive adjustments, residual or unmeasured confounding factors such as nutritional and physical activity habits cannot be ruled out. Second, liver biopsy was not performed in all participants in study 1 and 2. As our study involved individuals undergoing routine health examinations, the invasive nature of this procedure precluded its routine use. Alternatively, we used noninvasive assessment methods such as ultrasonography, transient elastography and MRI-PDFF. Third, the follow-up duration was relatively too short to adequately monitor the progression of advanced MASLD and its associated comorbidities. Fourth, due to the lack of insulin level measurements, the potential influence of insulin sensitivity on the association between Lp(a) and MASLD remains unexplored in this study. Finally, the measurement of Lp(a) was not performed using a uniform assay across all three cohorts. It may affect the direct comparability of Lp(a) concentration cut-offs between cohorts and underscores the need for assay standardization in future multi-center studies.

In conclusion, the novel founding in the present study indicates that serum Lp(a) levels decrease or increase with the progression or regression of MASLD. The findings from this study provide both mechanistic insights into MASLD development and clinically relevant implications for its early screening and prevention. More studies are necessary to verify the validity of our findings.

## Data Availability

The original contributions presented in the study are included in the article/Supplementary material, further inquiries can be directed to the corresponding authors.
