# Supplementary material for: Serum lipoprotein(a) levels are inversely associated with metabolic dysfunction-associated steatosis liver disease progression: two cross-sectional studies and a longitudinal study
Source: Front Nutr. 2026 Mar 17;13:1722393. doi: 10.3389/fnut.2026.1722393 (PMC13036134; doi:10.3389/fnut.2026.1722393)
Supplement: Supplementary file 1 [file Supplementary_file_1.docx]

Supplementary Table 1. The correlation between serum Lp(a) levels with the severity of hepatic steatosis by logistic regression analyses in study 1

|  | Model 1 | | Model 2 | |
| --- | --- | --- | --- | --- |
|  | OR(95%CI) | *P* | OR(95%CI) | *P* |
| Non-MASLD | Reference |  | Reference |  |
| mild MASLD | 0.802(0.721-0.893) | <0.001 | 0.949(0.845-1.066) | 0.381 |
| moderate MASLD | 0.655(0.606-0.707) | <0.001 | 0.848(0.773-0.930) | <0.001 |
| severe MASLD | 0.437(0.341-0.558) | <0.001 | 0.624(0.482-0.809) | <0.001 |

Model 1: unadjusted

Model 2: adjustment for age, sex, smoking, BMI, WBCC, monocyte count, neutrophil count, hypertension, diabetes, dyslipidemia, and serum uric acid

MASLD, metabolic dysfunction-associated steatotic liver disease; Lp(a), lipoprotein (a); BMI, body mass index ; WBCC, white blood cell count ; OR, odds ratio; CI , confidence intervals.

Supplementary Table 2. The correlation between serum Lp(a) levels with the severity of liver fibrosis by logistic regression analyses in study 1

|  | Model 1 |  | Model 2 |  |
| --- | --- | --- | --- | --- |
|  | OR(95%CI) | *P* | OR(95%CI) | *P* |
| F0-F1 | Reference |  | Reference |  |
| F2 | 0.739(0.661-0.826) | <0.001 | 0.806(0.717-0.907) | <0.001 |
| F3 | 0.607(0.502-0.736) | <0.001 | 0.671 (0.547-0.823) | <0.001 |
| F4 | 0.405(0.309-0.531) | <0.001 | 0.434(0.324-0.583) | <0.001 |

Model 1: unadjusted

Model 2: adjustment for age, sex, smoking, BMI, WBCC, monocyte count, neutrophil count, hypertension, diabetes, dyslipidemia, and serum uric acid

Lp(a), lipoprotein (a); BMI, body mass index ; WBCC, white blood cell count ; OR, odds ratio; CI , confidence intervals.

Supplementary Table 3. Baseline characteristics of individuals with or without MASLD in study 3

|  | Non‐MASLD (n=4512) | MASLD  (n=1425) | *P* |
| --- | --- | --- | --- |
| Age(years) | 54.90±7.57 | 55.23±7.36 | 0.141 |
| Male (n, %) | 1827(40.5) | 758(53.2) | <0.001 |
| Diabetes (n,%) | 100(2.2) | 88(6.2) | <0.001 |
| Hypertension (n,%) | 1699(37.7) | 730(51.2) | <0.001 |
| Dyslipidemia (n,%) | 1857(41.2) | 888(62.3) | <0.001 |
| BMI (kg/m2) | 25.59±3.72 | 29.37±4.35 | <0.001 |
| SBP (mmHg) | 135.31±18.71 | 140.33±17.31 | <0.001 |
| DBP (mmHg) | 80.19±10.26 | 84.34±9.91 | <0.001 |
| FPG (mmol/L) | 4.91±0.77 | 5.11±1.01 | <0.001 |
| TC (mmol/L) | 5.78±1.02 | 5.91±1.07 | <0.001 |
| TG (mmol/L) | 1.47±0.82 | 2.16±1.13 | <0.001 |
| LDL-C(mmol/L) | 3.60±0.78 | 3.80±0.81 | <0.001 |
| HDL-C(mmol/L) | 1.54±0.37 | 1.31±0.30 | <0.001 |
| Uric acid (mmol/l) | 286.94±72.35 | 335.12±75.160 | <0.001 |
| ALT (U/L) | 17.79 (14.04-23.06) | 24.81 (18.61-33.85) | <0.001 |
| AST (U/L) | 23.40 (20.40-27.20) | 25.40(21.90-30.20) | <0.001 |
| GGT (U/L) | 21.80 (16.20-31.88) | 31.70(22.50-46.90) | <0.001 |
| Lp(a) (mg/dL) | 9.86 (4.52-28.98) | 8.47 (3.90-28.40) | 0.006 |

Values are presented as mean ± standard deviation, n (%), or median (interquartile range)

BMI, body mass index ; SBP, systolic blood pressure ; DBP, diastolic blood pressure ;

WBCC, white blood cell count ; FPG, fasting plasma glucose ; TC, total cholesterol ; TG, triglyceride; LDL-C, low-density lipoprotein cholesterol ; HDL-C, high-density lipoprotein cholesterol ; ALT, alanine aminotransferase; AST, aspartate transaminase; GGT, gamma-glutamyl transpeptidase; MASLD, metabolic dysfunction-associated steatotic liver disease.


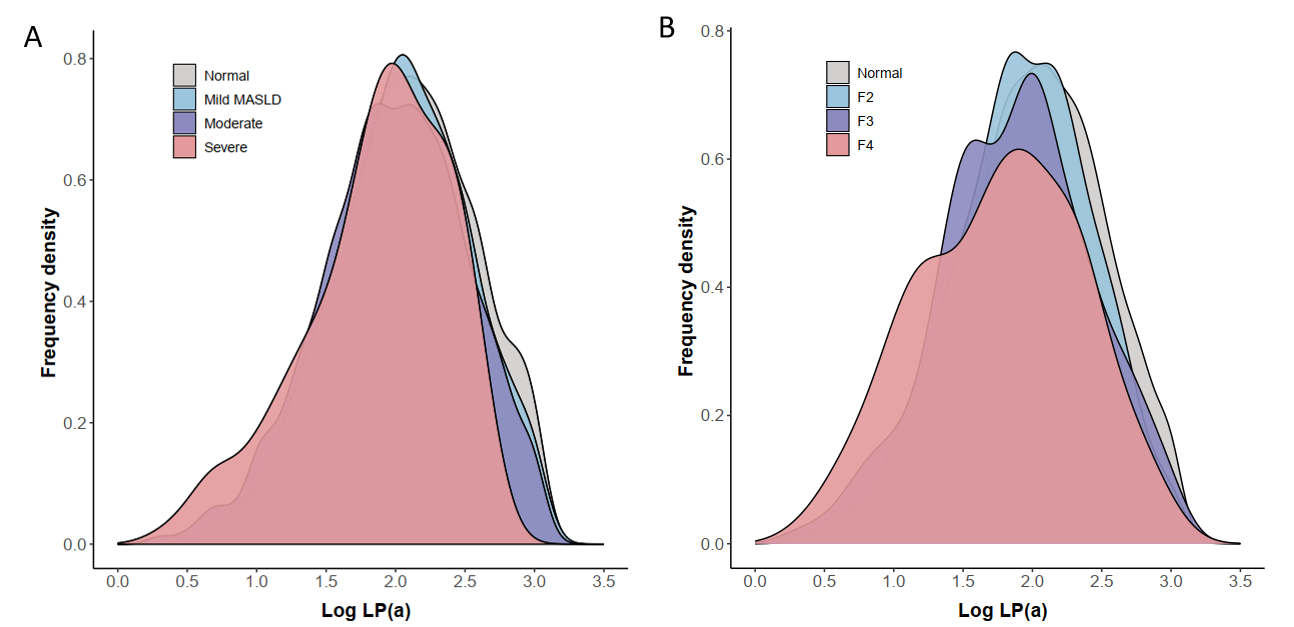


Supplementary Figure1. The distrubution of the log-transformed Lp(a) level stratified by the severity of hepatic steatosis and liver fibrosis in study 1.(A) the distrubution of the log-transformed Lp(a) level stratified by the severity of hepatic steatosis; (B) the distrubution of the log-transformed Lp(a) level stratified by the severity of liver fibrosis.
